# Racism Experience Among American Adults During COVID-19: A Mixed-Methods Study

**DOI:** 10.1089/heq.2022.0070

**Published:** 2022-08-05

**Authors:** Dejun Su, Khalid Alshehri, Jessica Ern, Baojiang Chen, Liwei Chen, Zhuo Chen, Xuesong Han, Keyonna M. King, Hongmei Li, Jian Li, Yan Li, Tzeyu Michaud, Lu Shi, Athena K. Ramos, Ming Wen, Donglan Zhang

**Affiliations:** ^1^ Department of Health Promotion, College of Public Health, University of Nebraska Medical Center, Omaha, Nebraska, USA.; ^2^ Center for Reducing Health Disparities, Department of Health Promotion, College of Public Health, University of Nebraska Medical Center, Omaha, Nebraska, USA.; ^3^ Department of Biostatistics and Data Science, School of Public Health, University of Texas Health Science Center at Houston, Austin, Texas, USA.; ^4^ Department of Epidemiology, Fielding School of Public Health, University of California Los Angeles, Los Angeles, California, USA.; ^5^ Department of Health Policy and Management, College of Public Health, University of Georgia, Athens, Georgia, USA.; ^6^ Surveillance and Health Equity Science, American Cancer Society, Atlanta, Georgia, USA.; ^7^ Department of Media, Journalism and Film, Miami University, Oxford, Ohio, USA.; ^8^ Department of Environmental Health Sciences, Fielding School of Public Health, School of Nursing, University of California Los Angeles, Los Angeles, California, USA.; ^9^ Department of Population Health Science and Policy, Icahn School of Medicine at Mount Sinai, New York, New York, USA.; ^10^ Department of Public Health Sciences, Clemson University, Clemson, South Carolina, USA.; ^11^ Department of Sociology, University of Utah, Salt Lake City, Utah, USA.; ^12^ Division of Health Services Research, Department of Foundations of Medicine, New York University Long Island School of Medicine, Mineola, New York, USA.

**Keywords:** racism experience, correlates, coping with racism, COVID-19

## Abstract

**Purpose:**

Despite escalating racism in the United States during COVID-19, few studies have identified correlates of racism experience among Americans using nationally representative data. This study seeks to quantitatively identify correlates of racism experience and qualitatively categorize racism experience and its coping using nationally representative survey data.

**Methods:**

Based on data from the “Health, Ethnicity and Pandemic Survey” (*N*=2,506), a nationally representative survey conducted in October 2020, multivariable logistic regression was estimated to examine the association between self-reported racism experience and selected correlates. Thematic analysis was conducted to qualitatively classify types of racism experience and related coping strategies.

**Results:**

When asked whether they had been discriminated or unfairly treated during COVID-19 because of their racial/ethnic background, 19% non-Hispanic Asian and Black respondents said yes, followed by 15% among Hispanics and 3% among non-Hispanic Whites. Besides significant correlates of racism experience identified at the individual and household level, three contextual factors at the neighborhood or state level were associated with lower odds of racism experience, including living in a blue state (adjusted odds ratio [AOR]=0.69, 95% confidence interval [CI]: 0.50–0.95; reference category: red state), living in the top third of the neighborhoods in the sample in terms of racial diversity (AOR=0.65%, 95% CI: 0.42–0.99; reference: bottom third), and coming from neighborhoods with a median population age of 35–39 (AOR=0.67, 95% CI: 0.46–0.98; reference: younger than 35). Prevailing coping strategies against experienced racism included social avoidance, direct confrontation, seeking social and religious support, resorting to hobbies for relief, and taking legal actions.

**Conclusion:**

Racism experience is not only correlated with factors at individual level, it is also associated with contextual factors such as political climate, neighborhood diversity, and population age structure. Future efforts in supporting victims of racism might be more cost-effective by focusing on the identified vulnerable groups and related contextual factors.

## Introduction

By the end of 2021, COVID-19 had claimed >800,000 lives in the United States, making it the deadliest pandemic in the country's history.^1^ Racial and ethnic minority Americans bear a disproportionate burden of COVID-19 primarily owing to social and structural determinants of health, which include disadvantages in physical and neighborhood environments (e.g., crowded living conditions, limited access to transportation), essential work in sectors with high exposure to COVID-19 (e.g., factories, farms, food processing plants, grocery stores, and public transportation), inadequate access to affordable and culturally responsive health services, poverty, and lack of access to quality education.^2^ An important factor underlying COVID-19 disparities is racism, which can be defined as “beliefs, attitudes, institutional arrangements, and acts that tend to denigrate individuals or groups because of phenotypic characteristics or ethnic group affiliation.”^3^

Based on this definition, racism can manifest at both the macro- and microlevel. At the macrolevel, institutional or structural racism fosters and maintains racial discrimination through mutually reinforcing inequitable systems or sectors, including but not limited to education, employment, credit markets, health care, housing, and criminal justice.^4^ At the microlevel, racism is primarily epitomized by prejudices against certain racial or ethnic groups and related manifestations in interpersonal relationships, such as racial microaggression^5,6^ and hate crimes based on race, ethnicity, and national origin.^7^

For many Americans, especially racial and ethnic minorities, the fight against COVID-19 has concurred with a personal struggle to cope with intensified racism and xenophobia in the country. According to a national poll conducted in 2020 by the Pew Research Center, 26% of Asian Americans feared that someone might threaten or physically attack them because of their race or ethnicity, followed by 20% among African Americans and 10% among the Hispanic population.^8^ More recent results from another survey conducted by the Pew Research Center in 2021 revealed that the percentage of Asian Americans who feared that someone might threaten or physically attack them increased to 32%, followed by 21% among African Americans and 16% among Hispanics.^9^

These perceptions and their changes over time were corroborated by recent trends in hate crimes against Asian Americans and African Americans. In 2020, a total of 5,227 race/ethnicity/ancestry-based hate crimes were reported to the Federal Bureau of Investigation (FBI), a 32% increase from 2019.^10^ The corresponding increase in anti-Asian hate crimes during the same period was 77%, followed by an increase of 49% in anti-Black hate crimes.^10^

Consistent with the national trend on the recent escalation of racism and hate crimes against racial and ethnic minorities, a growing number of studies documented the prevalence of reported discrimination at the individual level and its association with anxiety, psychological distress, and other mental health issues among racial and ethnic minorities during the COVID-19 pandemic.^11–18^ Despite the importance and timeliness of these findings, three limitations are noteworthy. First, none of these studies were based on nationally representative samples, and the modest sample size in most of these studies limits the generalizability of their findings. Second, extant research provides little evidence on how contextual factors—such as neighborhood ethnic composition, economic status, and political climate—might shape the perception of racial discrimination during the pandemic. Third, it remains unclear and underexplored how Americans coped with discrimination during the COVID-19 pandemic. Identifying prevailing coping strategies is instrumental for developing future interventions to mitigate the negative effect of racial discrimination.

Based on analysis of quantitative and qualitative data collected from a recent, nationally representative sample of adult Americans, this study has two aims: (1) to identify correlates of racism experience at the individual, household, and neighborhood levels; and (2) to classify and qualitatively describe the specific types of racism experience and actions taken to cope with the discrimination.

## Methods

### Data

The primary source of data used in this study came from the “Health, Ethnicity, and Pandemic (HEAP) Survey” conducted in October 2020 by the National Opinion Research Center (NORC) at the University of Chicago. The main purpose of the survey was to assess changes in physical and mental health, health behavior, health care access, and social determinants of health including exposure to racism during the pandemic, with oversampling of racial and ethnic minorities. The initial sample was randomly drawn from NORC's AmeriSpeak Panel, a probability-based panel designed to be representative of the US noninstitutionalized population.^19^ Detailed information regarding the panel recruitment and selection methodology can be found in previous publications using HEAP data.^20–23^

The HEAP survey was delivered in English and Spanish through the internet or telephone based on the preferences of respondents. NORC implemented stratified sampling and poststratification weighting procedures to ensure the study sample was nationally representative of US adults (age ≥18 years). The study was approved by the Institutional Review Board at the NORC. A total of 2,709 respondents participated in the survey.

The HEAP data contained information on residential locations such as zip codes, counties, and the states where respondents live, which allows for the merging of HEAP data with three sources of neighborhood- or state-level data based on related geographic identifiers. The first source is the 2018 Social Determinants of Health variables at the zip code level released by the Agency for Health care Research and Quality^4^ where two variables, population median age and median household income at the zip code level were merged with HEAP.

The second source of neighborhood data came from the 2019 American Community Survey, whereby information on ethnic composition at the zip code level was used to calculate an index (ethnic heterogeneity index) denoting the level of ethnic heterogeneity in the neighborhood.^24^ The third source of neighborhood-level data was the Electoral College results from the 2020 Presidential Election to denote the general political ideology in the state where the respondents live (blue vs. red states).^25^ The final merged sample used in this study consisted of 2,506 respondents with complete information on selected variables at the individual, household, and neighborhood level.

### Measures

#### Outcome measure

The outcome measure on racism experience was based on the survey question: “Have you personally experienced any discrimination or unfair treatment because of your racial or ethnic background during the COVID-19 pandemic?” (Yes, No). For the 369 respondents who answered “yes” to this question, they were further asked to qualitatively describe: (1) the kind of discrimination or unfair treatment they had experienced, and (2) what they did to cope with the experienced discrimination or unfair treatment based on two open-ended questions. These qualitative data were available for 300 respondents.

#### Covariates at the individual level

Demographics at the individual level include age groups (18–29, 30–49, 50–69, 70+), gender (male, female), race/ethnicity (non-Hispanic White, Hispanic, non-Hispanic Black, non-Hispanic Asian, other), nativity (born in the United States vs. foreign countries), and marital status (married, unmarried). Socioeconomic status variables included education level (less than high school, high school, vocational/tech school/some college, bachelor's degree or above) and employment status (employed, retired, unemployed). Religiosity was partially measured by the frequency of attending church, synagogue, or other religious meetings in person or online (more than once a week, once a week, once or twice a month, a few times a year, seldom, never).

The health status of respondents was characterized by two variables, including self-rated health (excellent, very good, good, fair, poor) and self-assessment of changes in bodyweight during the pandemic (no change, gained weight, lost weight). Two COVID-19-related health behaviors included the practice of physical distancing in public spaces (always, sometimes, never) and wearing masks in public spaces (always, sometimes, never).

#### Covariates at the household level

The economic status of the household was primarily measured by two variables: annual household income in the last year ($24,999 or less, $25,000-$59,999, $60,000-$149,999, $150,000 or more) and homeownership (owned, rented, and occupied without paying rent). A related third variable at the household level denoted whether there was internet access (yes, no).

#### Covariates at the neighborhood/state level

Neighborhood demographics were characterized by population median age (34 years or younger, 35–39, 40 years or older) and the ethnic heterogeneity index (low, middle, high). This index is a measure that takes a value between 0 (of minimum heterogeneity) and 1 (of maximum heterogeneity) to indicate the level of racial and ethnic diversity of the population at the neighborhood level, and the calculation of the index value was based on a prespecified formula.^26,27^ For ease of interpretation, the neighborhood-level factors of population median age, median household income, and the ethnic heterogeneity index were categorized into three groups based on roughly similar proportions of observations to represent the bottom third, middle third, and top third of cases. Based on the results of the 2020 Presidential Election, states were categorized into blue or red states to denote the general political ideology.

### Statistical analysis

The analytical section of the study started with a comparison between respondents who reported experience of racial discrimination and those otherwise in terms of selected covariates at the individual, household, and neighborhood level. *p*-Values based on chi-square tests were estimated to indicate if a bivariate association was found. This was followed by logistic regression to examine the association between racism experience and selected covariates. All statistical analyses were performed with poststratification weighting to account for the complex survey design and sampling procedures. Values of *p*<0.05 based on two-tailed tests were considered statistically significant. SAS 9.4 software was used for data merging, cleaning, coding, and analysis.

For the qualitative data on types of discrimination experienced and coping strategies adopted, two researchers in the study team first independently conducted an inductive thematic analysis to systematically code the data to identify categories and themes.^28^ The two researchers then compared their results and resolved inconsistencies through discussions. Cohen's kappa coefficients were calculated to evaluate the consistency between the two researchers, which showed a value of 0.94 for discrimination types and 0.90 for coping strategies, indicating a high level of consistency.

## Results

As given in Table 1, in total, ∼9% of the weighted sample reported experiencing racial discrimination during the COVID-19 pandemic. The prevalence of racism experience, however, differed across the four major racial and ethnic groups, with non-Hispanic Blacks and non-Hispanic Asians, respectively, showing the highest rate of ∼19%, followed by 15% among Hispanics, and 3% among non-Hispanic Whites. Based on the bivariate associations, being young, male, foreign-born, unmarried, having low education, having fair or poor self-rated health, gaining weight based on self-assessment, attending religious services more often, being independent in party affiliation, not always wearing masks in public, living in households with economic disadvantages, and living in neighborhoods with younger populations were more likely to experience discrimination.

**Table 1.** Comparison Between American Adults Who Reported Experience of Racial Discrimination and Those Otherwise During the COVID-19 Pandemic: the 2020 Health, Ethnicity, and Pandemic Study with Poststratification Weighting

Variables	Experience of discrimination during COVID-19			
	Weighted *n*	Yes %	No %	*p*
	2458	9.00	91.00	—
Individual-level factors				
Age, years				<0.001
18–29	471	10.84	89.16	
30–49	834	12.34	87.66	
50–69	824	7.43	92.74	
70+	329	2.23	97.77	
Gender				0.003
Male	1191	10.75	89.25	
Female	1268	7.35	92.65	
Race/ethnicity				<0.001
Non-Hispanic White	1486	3.01	96.99	
Hispanic	425	15.01	84.99	
Non-Hispanic Black	300	19.46	80.54	
Non-Hispanic Asian	152	18.68	81.32	
Other	96	27.05	72.95	
Country of birth				0.006
In the United States	2195	8.45	91.55	
Outside the United States	263	13.58	86.42	
Marital status				<0.001
Unmarried	1202	11.97	88.03	
Married	1256	6.15	93.85	
Education level				<0.001
Less than high school	245	17.38	82.62	
High school graduate or equivalent	677	9.91	90.09	
Vocational/tech school/some college	701	9.65	90.35	
Bachelor's degree or above	835	5.25	94.75	
Employment status				<0.001
Employed	1416	8.80	91.20	
Retired	465	4.44	95.56	
Unemployed	578	13.14	86.86	
Religious attendance				0.019
More than once a week/once a week	671	9.11	90.89	
Once or twice a month/a few times a year	490	12.04	87.96	
Seldom/never	1298	7.79	92.21	
Political party				0.024
Democrat or lean democrat	1268	9.58	90.42	
Independent	313	11.90	88.10	
Republican or lean republican	877	7.12	92.88	
Self-rated health				0.005
Excellent/very good/good	2125	8.36	91.64	
Fair/poor	334	13.07	86.93	
Self-assessment of bodyweight changes during COVID-19				<0.001
No change	921	6.17	93.83	
Gained weight	1035	11.59	88.41	
Lost weight	502	8.85	91.15	
Practice physical distancing in public during the pandemic				0.563
Always	1707	8.78	91.22	
Sometimes/never	752	9.50	90.50	
Wearing a mask in public during the pandemic				<0.001
Always	2050	8.10	91.90	
Sometimes/never	408	13.50	86.50	
Household-level factors				
Household income				<0.001
$24,999 or less	473	15.71	84.29	
$25,000–$59,999	742	10.02	89.98	
$60,000–$149,999	1009	5.94	94.06	
$150,000 or more	234	5.36	94.64	
Home ownership				<0.001
Owned	1618	6.12	93.88	
Rented	767	14.34	85.66	
Occupied without paying rent	74	16.50	83.50	
Household internet access				<0.001
Yes	2216	8.16	91.84	
No	243	16.66	83.34	
Neighborhood-level factors				
General political ideology in the state (based on the 2020 presidential election)				0.061
Blue states	1409	8.06	91.94	
Red states	1049	10.25	89.75	
Ethnic heterogeneity index				0.004
Low (0–0.39)	1189	7.20	92.80	
Middle (0.4–0.59)	684	11.72	88.28	
High (0.6 or above)	585	9.47	90.53	
Population median age				<0.001
34 or younger	636	12.72	87.28	
35–39	832	8.50	91.50	
40 or older	990	7.03	92.97	
Median household income				0.013
Low (<$49,500)	798	11.44	88.56	
Middle ($49,500–$70,500)	944	7.93	92.07	
High (>$70,500)	716	7.68	92.32	

Some of the aforementioned bivariate associations were confirmed in the logistic regression when adjusting for the effects of other covariates (Table 2). At the individual level, relative to the age group of 18–29 years old, respondents of ages 30–49 were more likely to report experiencing racial discrimination (odds ratio [OR]=1.90, 95% confidence interval [CI]: 1.25–2.91), whereas respondents aged 70 or older were less likely to report discrimination (OR=0.36, 95% CI: 0.13–0.99). Women were much less likely than men to report racial discrimination (OR=0.54, 95% CI: 0.39–0.74). Again, racial and ethnic minorities were much more likely than non-Hispanic Whites to report racial discrimination during the pandemic. For example, relative to non-Hispanic White respondents, the odds for non-Hispanic Asian respondents to report experiencing discrimination during the pandemic was much higher (OR=13.80, 95% CI: 7.36–25.89). A higher level of educational attainment was generally associated with reduced odds of reporting racial discrimination.

**Table 2.** Multiple Logistic Regression on Racism Experience Among American Adults Amid the COVID-19 Pandemic: the 2020 Health, Ethnicity, and Pandemic Study (with Poststratification Weighting, *N*=2458)

Variables	OR	95% CI
Individual-level factors		
Age, years		
18–29	Reference	
30–49	1.90^**^	1.25–2.91
50–69	1.24	0.77–2.01
70 or more	0.36^*^	0.13–0.99
Gender		
Male	Reference	
Female	0.54^***^	0.39–0.74
Race/ethnicity		
Non-Hispanic White	Reference	
Hispanic	4.71^***^	2.92,7.61
Non-Hispanic Asian	13.80^***^	7.36–25.89
Non-Hispanic Black	7.50^***^	4.52–12.43
Other	16.20^***^	8.65–30.33
Country of birth		
In the United States	Reference	
Outside the United States	0.86	0.54–1.36
Marital status		
Unmarried	Reference	
Married	0.71	0.50–1.01
Education level		
Less than high school	Reference	
High school graduate or equivalent	0.60^*^	0.38–0.97
Vocational/tech school/some college	0.70	0.43–1.15
Bachelor's degree or above	0.34^***^	0.19–0.61
Employment		
Employed	Reference	
Retired	1.57	0.81–3.05
Unemployed	1.20	0.83–1.73
Religious attendance		
More than once a week/once a week	Reference	
Once or twice a month/A few times a year	0.96	0.62–1.47
Seldom/never	0.84	0.57–1.23
Political party		
Democrat or lean democrat	Reference	
Independent	0.79	0.50–1.23
Republican or lean republican	1.02	0.68–1.52
Self-rated health		
Excellent/very good/good	Reference	
Fair/poor	1.11	0.74–1.68
Self-assessment of bodyweight changes during the pandemic		
No change	Reference	
Gained weight	1.53^*^	1.05–2.21
Lost weight	1.28	0.82–2.02
Physical distancing in public during the pandemic		
Always	Reference	
Sometimes/never	0.91	0.61–1.34
Wearing a mask in public during the pandemic		
Always	Reference	
Sometimes/never	2.66^***^	1.69–4.19
Household-level factors		
Household income		
$24,999 or less	Reference	
$25,000–$59,999	1.01	0.67–1.52
$60,000–$149,999	0.78	0.48–1.27
$150,000 or more	0.98	0.46–2.10
Home ownership		
Owned or being bought by you or someone in your household	Reference	
Rented for cash	1.38	0.98–1.95
Occupied without payment of cash rent	1.58	0.75–3.34
Household internet access		
Yes	Reference	
No	1.65^*^	1.07–2.54
Neighborhood/state-level factors		
General political ideology in the state (based on 2020 presidential election)		
Red states	Reference	
Blue states	0.69^*^	0.50–0.95
Ethnic heterogeneity index		
Low	Reference	
Middle	0.93	0.64–1.36
High	0.65^*^	0.42–0.99
Population median age, years		
34 or younger	Reference	
35–39	0.67^*^	0.46–0.98
40 or older	0.78	0.51–1.17
Median household income		
Low	Reference	
Middle	1.28	0.88–1.86
High	1.36	0.88–2.12

Notes: ^*^p<0.05; ^**^p<0.01; ^***^p<0.001.

CI, confidence interval; OR, odds ratio.

Relative to those who reported no change in bodyweight during the pandemic, respondents who believed they had gained weight were more likely to report discrimination (OR=1.53, 95% CI: 1.05–1.21). Another interesting finding concerned mask-wearing in public. Compared with respondents who reported that they always wore a mask in public during the pandemic, the odds for respondents who never or seldom wore a mask in public to report racial discrimination were much higher (OR=2.66, 95% CI: 1.69–4.19).

The only variable at the household level that showed a significant association with reported discrimination was internet access. Respondents from households with no internet access were more likely than their counterparts with internet access at home to report racism experience during the pandemic (OR=1.65, 95% CI: 1.07–2.54).

Respondents living in a blue state at the time of the survey were 31% less likely to report racism experience compared with those from a red state (OR=0.69, 95% CI: 0.50–0.95). Relative to those living in the least diverse neighborhoods, the odds for respondents from highly diverse neighborhoods to report racism experience was 35% less likely (OR=0.65, 95% CI: 0.42–0.99). The age structure of the neighborhood also tended to make a difference. Neighborhoods with population median ages ranging between 35 and 39 years were 33% less likely to report racial discrimination compared with those living in neighborhoods with median ages of 34 years or younger (OR=0.67, 95% CI: 0.46–0.98).

The specific types of racial discrimination experienced by respondents in the survey are given in Table 3, whereby three themes and their subthemes, as well as the sample quotes corresponding to each of the subthemes are presented. Although the first two themes, “verbal accusation/personal attack” and “viewed/treated differently” were mainly based on personal, direct experience, the third theme “perception of general racism against one's ethnic background” was more related to prevailing racism at the societal level, as exemplified by blaming Asians for the pandemic, political leaders using racist labels for COVID-19, and racial slurs or racially motivated accusations in social media or the internet.

**Table 3.** A Typology of Reported Experience of Racial Discrimination Among American Adults During the COVID-19 Pandemic: the 2020 Health, Ethnicity, and Pandemic Study

Theme	Subtheme	Sample quotes
Verbal accusations/personal attack	Comments based on nationality, race or ethnicity	“I was in the market when a person walked by and was muttering racial epithets while accusing me of having caused the pandemic.” “While taking my regular walk and jogging in my neighborhood, a white person passed me on his bike, and yelled at me.” “Racial slurs regarding alleged source of COVID-19 and phrases to go back where you came from”
	Comments based on appearance	“Wearing masks that fog up my glasses. Been called foggy four eyes due to the mask.” “Mocked for Appearance”
	Other insulting comments	“Been told I was a dumb”
Viewed/treated differently	Social avoidance	“An old lady looked at me and noticed I'm Asian and she overly tried to stay away from me with giving me a dirty look when we passed each other at the hallway at the clinic.” “Going to the grocery stores people tend to move away from me because I'm black and they think I will give them the virus because on the news they said more blacks are infected with the virus.” “Being ignored by a white customer.”
	Denial of services	“They refused to let me rent a car.” “Not taking my services.”
	Career or workplace discrimination	“No promotion for Asian.” “Being let go from an employer.” “I was passed up for promotion on my former job. I was better qualified than the person they promoted.”
	Spoken language prejudice	“Discriminated because I could not speak Spanish.” “A man told me to get out of his store because I didn't speak English right.”
Perception of general racism against one's ethnic background	Nationality-based prejudice/pandemic blame	“Asians cause the pandemic.” “Constantly hearing President Trump constantly calling it the ‘China Virus' and constantly blaming China for the pandemic.” “Trump calling the pandemic kung flu and claiming it came from China.” “Because Trump keeps using the Chinese Flu instead of using COVID-19.”
	Internet or media judgments	“Racial slurs online.” “I am a white male. All the media, news, even work state that I am the most evil person alive. It gets very frustrating that is how I am made to feel. How about treat all people with respect and dignity.”

Coping strategies adopted by respondents who reported experiencing racism during the pandemic were classified into six categories, as given in Table 4. The most commonly adopted coping strategy was social avoidance, followed by direct confrontation, seeking social and religious support, resorting to hobbies for relief, and taking legal actions.

**Table 4.** A Typology of Coping Strategies Against Experienced Racial Discrimination Among American Adults During the COVID-19 Pandemic: the 2020 Health, Ethnicity, and Pandemic Study

Themes	Sample quotes
Avoidance or simply ignoring (*n*=37)	“I got my son and we left.” “Took breaths and ignored them.” “Just being quiet and polite…” “I avoided the place.”
Direct confrontation (*n*=26)	“Speak up.” “I said no and denied the person service.” “I go off on them back, I do not stand down. If you disrespect me then I will treat you as so!” “Stand up for myself.” “I said you should be wearing one too.” “Report racism on social media via contacting admin on their platform. Having discussions about race and the political polarity that has caused more racism in the U.S.”
Seeking social and religious support (*n*=26)	“Pray for them and forgive them for they know not what they do.” “Talk about it with my family and learned how to shrug it off.” “Prayed for the people discriminating against me.” “Talked about it with friends and family.”
Resorting to hobbies for relief (*n*=18)	“I drink alcohol daily.” “I used my punching bag.” “Listen to loud music.” “I smoke marijuana.”
Taking legal actions (*n*=12)	“I've talk about the situation with my Counselor and was advised to put in my complaint with school district.” “Call the cops.” “Ignored the people and later reported them.” “Looked into a lawyer.”

## Discussion

Consistent with related findings from previous national assessments of racism experience during COVID-19,^8,9^ this study identified a substantial prevalence of racism experienced by American adults, with Asian and Black Americans more likely to report experiencing racial discrimination than other racial groups. Discussions of these findings cannot be separated from the historical context of October 2020 when the HEAP Survey was conducted. This was a unique moment in the history of the United States as the country was scrambling to respond to a historic pandemic amid rising tensions in racial relations, notably marked by countless protests throughout the nation against police brutality against African Americans and growing discrimination and hate crimes against Asian Americans.^10,29^ Meanwhile, the political polarization and mobilization right before the 2020 Presidential Election provided room for political ideology to shape the perception of racism and personal experience about it.^30^

One interesting and unique finding from this study concerned the significant differences between respondents from blue and red states in their odds of reporting racism experience. The observation that living in a blue state was associated with lower odds of racism experience than living in a red state pointed to the relevance of the overall political ideology and climate as an important contextual factor. Because Americans of racial and ethnic minority background overwhelmingly support Democrats,^31^ it is understandable that for many minority Americans who live in a red state, they might disagree or even feel frustrated with prevailing policies and regulations in the state. Such emotional somatization could become intensified given the political polarization under the Trump presidency. Because the majority of minority Americans live in blue states, the political and social isolation of minority Americans in red states could contribute to their perception of racism experience.

This study also identified racial and ethnic diversity in residential neighborhood as another significant contextual factor shaping racism experience. Respondents from more diverse communities turned out to be less likely to report experience of racial discrimination than those from less diverse communities. Americans from diverse communities may have grown accustomed to interacting with people of different racial and ethnic backgrounds in their community even before COVID-19, which makes them less likely to experience or perceive discrimination during the pandemic. On the contrary, for residents from more racially homogenous communities, the lack of interaction with, and presumably understanding of, people from a different racial or ethnic background could make them more likely to heed to prevailing biases and stereotypes to make sense of the pandemic and evolving racial relationships.

Discrimination in housing and credit markets contributes to residential segregation, which in turn can lead to concentrated poverty, poor health outcomes, and lack of access to educational and employment opportunities among minority populations.^32^ Addressing residential segregation and its contributing factors should become an integral part of future efforts in eradicating structural and institutional racism.

Findings from this study revealed several other significant correlates of racism experience at the individual and household level, which included being male, in the age range of 30–49 years, having low education attainment, self-report weight gain, not always wearing a mask in public, and having no internet access at home. These findings point to the groups of people vulnerable to racism that future interventions might want to prioritize. Americans of ages 30–49 years constitute the bulk of the workforce and their more frequent interactions with people from diverse background at work might make them more likely than Americans of other age groups to experience racism. Some of the other correlates, such as low education and lack of access to internet services, underscored the relevance of socioeconomic status in explaining disparities in racism experience.

A plausible explanation for the observed association between self-report weight gain and racism experience was that, on the one hand, perceived discrimination might incur weight gain,^33^ yet, on the other hand, gaining weight itself can also trigger weight-based discrimination.^34^ As for the association between mask-wearing and racism experience, it is possible that some of the racism experience reported in the HEAP Survey might be owing to disputes revolving around complying with mask-wearing regulations in public spaces.

The qualitative data collected in HEAP provided further details on the types of reported discrimination. Although some of the brief quotes such as “Being let go from an employer” made it difficult to infer whether such an encounter constitutes racial discrimination based on prevailing definitions, there is less ambiguity when it came to the subtheme of nationality-based prejudices and pandemic blame. Asian respondents in the survey, especially those of Chinese descendance, were overwhelmingly offended by former President Trump's constant use of racial slurs to depict COVID-19.

Among respondents who indicated how they responded to the racial discrimination they had experienced, the most frequently adopted strategy was avoidance or simply ignoring the incidence. Whether and the extent to which this strategy and other identified coping strategies were effective in helping the respondents mitigate the harmful effect of discrimination remain to be evaluated. It would also be valuable to examine whether a combination of different coping strategies might be working better than resorting to a single coping strategy, and further identify specific strategies that are effective for coping with certain types of discrimination or for certain groups of people.

### Limitations of the study

The significance of the main findings from this study should be assessed in light of several limitations. First, the qualitative data collected in this study were based on answers to two open-ended questions in a survey. The depth and richness of the information certainly would not be comparable to collecting the same data through personal interviews or focus group discussions.

The second limitation concerns the difficulty in inferring causality when collecting data from a cross-sectional survey like HEAP. For example, it becomes challenging to disentangle the association between gaining weight and racism experience and infer which one is the cause and which one is the consequence.

Third, because the HEAP survey was conducted at a unique historical moment in the United States, caution should be taken before generalizing findings from this study to assess racism experience and its correlates in other times.

Finally, despite the revealed relevance of neighborhood-level factors in accounting for racism experience, the modest sample size of HEAP makes it challenging to conduct rigorous multilevel analysis (e.g., Hierarchical Linear Modeling) to more reliably assess this association after adjusting for the effect of related factors at the individual level, which can be a step for future research along this line.

## Conclusions

Cumulative evidence from a growing body of research has documented the negative influence of experiencing racism on psychosocial and mental health.^11–18^ Based on survey data from a nationally representative sample, findings from this study highlighted the complexities of factors associated with racism experience amid the COVID pandemic. In particular, Americans who were racial and ethnic minorities, male, of ages 30–49 years, having an education of less than a high school, gaining weight, not always wearing masks in public, living in households without internet access, coming from racially more homogenous neighborhoods, and living in red states were more likely to report racism experience. Future efforts in supporting victims of racism and mitigating its health impact could become more cost-effective by purposefully engaging the identified vulnerable groups. There is also a need to build upon the reported, various coping strategies in response to racism experience and to assess their effectiveness in mitigating the negative influence of racism experience on psychosocial wellbeing and mental health.

## Abbreviations Used

AOR: adjusted odds ratio
CI: confidence interval
FBI: Federal Bureau of Investigation
HEAP: Health, Ethnicity, and Pandemic
NORC: National Opinion Research Center
OR: odds ratio
